# Obstetrics

**Published:** 1907-06

**Authors:** Walter C. Swayne


### OBSTETRICS.

The prevention and treatment of **post-partum hemorrhage** is a subject of never-failing interest to the practitioner, and in view of the sudden and frequently fatal catastrophe which may ensue as the result of its occurrence, every attempt to suggest new or improved methods for either its prevention or cure demands careful examination.

Stanmore Bishop,[1] as the result of an article on this subject, has been the cause of a considerable correspondence. In his original article he produces many arguments for the employment of a method certainly not new, and sufficiently well known, although not widely taught or practised as a primary method of dealing with this complication, namely compression of the abdominal aorta.

He gives a résumé of the different causes of post-partum hemorrhage as generally taught, under the following headings, namely primary external atonic, primary concealed atonic, secondary external traumatic and secondary concealed traumatic, and states that while the attendant is endeavouring to satisfy himself under which of these heads the particular case falls, the patient is being rapidly exhausted by bleeding.

He also states that in addition to the delay while the exact cause is being mentally decided on, a prolonged search for tears of the viscera, uterus or vagina is necessary, which has to be carried out in the midst of a torrent of blood, the clots from which obscure everything, and only after this exploration can anyone feel certain that the cause is not traumatic ; and all through this tedious and difficult exploration the patient's vitality is rapidly becoming less.

Next, he states the student is taught that there are fourteen distinct causes of atonic post-partum hemorrhage, the first of these being improper management of the third stage, and describes the student as often exciting irregular and ineffectual uterus contractions by squeezing the uterus during its intervals of partial relaxation, and then removing the placenta by the hand introduced into the vagina or uterus.

After giving a list of the fourteen causes of atonic post-partum hemorrhage, he proceeds to eliminate a large number, such as hæmophilia, which is, as he states, rare, inversion of the uterus, which does not occur if the third stage is properly managed, and adherent or retained placenta, as regards which he considers that any attempt at removal whilst bleeding is going on is to court disaster, since it is certain to be attempted with unsterilised hands, while the presence of the placenta in the uterus makes no difference to the really effective measures which will arrest the bleeding.

[1] *Practitioner*, 1906, lxxvii. 145.

He groups all the remaining causes under the heading of " tired uterus," and points out that it is useless to attempt to stimulate contraction in a uterus that is relaxed, because it is " tired," and so has lost temporarily the power of contraction.

He makes much of the difficulty of explaining what is meant by uterine " retraction." The difficulties of explanation are not great, and, after all, quibbling over the precise explanation of a term is not of any great assistance to the seeker after truth.

He denies unhesitatingly that uterine contraction—to the securing which all the energies of the attendant should be directed—is the thing which should be aimed at, and states that the truth is exactly the opposite. " We must not look to uterine contraction to stop the hemorrhage, but to cessation of the hemorrhage in order to permit once more of contraction of the uterus."

He then states that there are six various measures given by writers on midwifery by means of which uterine contraction may be brought about, and asks, " Do the advisers of these various measures believe in any one of them ? No. We are to ' try ' them all, one after the other, and no one for any length of time." They are expected to fail, as to each one is appended the phrase, " If this fail." All having been tried, thrombosis, produced by the action of perchloride of iron or other styptic, is the last resource. Finally, having proved the uselessness of all the measures advised, he draws the attention of readers to the fact that hemorrhage from veins can be stopped by elevating the bleeding part, and from arteries by compressing their parent trunks, and that consequently elevation of the pelvis will stop such uterine hemorrhage as comes from uterine veins, and pressure on the aorta such as comes from uterine arteries.

He, in consequence, advocates the raising of the foot of the bed by pushing a table beneath it, or placing the feet of the bed on two chairs, in order to cope with the venous, and compressing the aorta in order to stop the arterial hemorrhage.

Having in this way checked the immediate outflow of blood, the uterus, directly it shows signs of returning vitality, is assisted to expel contained clots, and the compression of the aorta handed over to the nurse, while the surgeon proceeds to sterilise his hands for the purpose of removing adherent placenta, or searching for lacerations.

He argues that the circulation through the ovarian arteries not being cut off is sufficient to preserve the contractility of the uterus, and keep it sufficiently supplied with blood to ensure its muscular fibre retaining its vitality. He gives, in support of his contention, the instance of a thigh cut off by a circular saw, and asks, " Would the surgeon in such a case, supposing his usual appliances absent, squirt hot water at the stump or apply perchloride of iron " ?

He insists that a combination of the two methods he suggests, namely elevation of the pelvis and compression of the aorta, are the only true ways of combating post-partum hemorrhage, and that these two methods in combination must be persisted in, the latter for three hours if necessary, and the former for twelve.

Gordon Fitzgerald[1] protests against the condemnation of well-tried methods on the experience of one man, and against that of many others who have proved the efficiency of the methods they advocate *when properly applied.* He dissents from Mr. Stanmore Bishop's description of a typical case of such hemorrhage as occurring in a healthy young primipara, who has had satisfactory first and second stages of her labour, and dies of *acute primary atonic post-partum hemorrhage, primary inertia.*

He states that the forces which naturally control post-partum hemorrhage are contraction, retraction, and clotting.

He points out that neither active contraction nor clotting will permanently check post-partum hemorrhage in the absence of retraction, which he describes as that plastic force which moulds the uterus to its contents. He admits that retraction is aided by contraction, but he has as yet seen no convincing proof that it is dependent on contraction, and cannot come into action in its absence.

He allows that compression of the aorta will cause the cessation of post-partum hemorrhage, but considers that it is difficult to carry out effectively, except in hospital practice, where there are numerous assistants. He doubts the influence of elevation of the pelvis on hemorrhage coming from the veins, especially if the aorta is compressed. He relies mainly on prophylaxis, advising that hæmophilia, if it be present—and it is well known that it is a very rare condition in the female—be combated in advance by the administration of arsenic, strychnine and chloride of calcium.

Protraction of the first and second stages is to be avoided, and the third stage as prolonged as is consistent with the safety of the patient. All preparations for the treatment of post-partum hemorrhage should be made in advance.

Prolongation of the second stage should be avoided by the early use of forceps, but prolongation of the first stage is comparatively unimportant.

The indications for termination of the second stage will be found in the conditions of the patient's pulse and temperature In cases of hydramnios and over-distension from twins, especially in multiparæ, the emptying of the uterus should be gradual, and plenty of time allowed in the second stage; by early puncture of the membranes high up in the first condition, and allowing of considerable delay between the birth of the two children in the second, so that the over-distended uterus, whose contractions are

[1] *Practitioner*, 1906, lxxvii, 652.

sure to be weak at first, may gather strength for its final efforts. In the case of multiparæ, a previous history of adherent placenta will prepare us for dealing with that condition.

He advises after thirty minutes waiting, if the slight hemorrhage which occurs with each contraction due to the expulsion of already effused blood does not diminish, the extraction of the placenta with the gloved hand.

The improper management ot the third stage is the great predisposing cause.

The immediate grasping of the uterus and expulsion of the placenta directly after the birth of the child is one of the most fertile causes. Unless there is undue hemorrhage, no efforts at expulsion should be practised unless the placenta is found to be in the vagina. That it is in the vagina can be recognised by drawing out all the loops of cord in the vagina, and then grasping the uterus and lifting it towards the ensiform cartilage, when, if the placenta is still in the uterus, the cord will be drawn back into the vagina.

Forcible and rapid attempts at expulsion result in irregular contractions, possibly imprisonment of the placenta in the uterus by hour-glass contraction, or detachment of portions of its maternal surface.

E. Hastings Tweedy[1] considers that Stanmore Bishop's statement that "post-partum hemorrhage is essentially a general practitioner's tragedy, and that from his own experience and that of others, cases do not occur in hospital," is unwarranted. He finds that the statistics of the Rotunda show that in 5,695 women there occurred fifty-six cases of post-partum hemorrhage, of which thirty-one were due to retained placenta, membranes, or clots mechanically preventing the closing down of the uterus, three were traumatic, and four of the secondary variety. Of the fifty-six cases, three died from supervening shock.

Compression of the abdominal aorta as a means of controlling hemorrhage has been taught and practised at the Rotunda for years, and is looked upon as a valuable temporary expedient.[2]

That retraction is independent of contraction is shown by the following arguments. The uterus does not commonly contract for about seven minutes after the birth of the child. If the prevention of hemorrhage depended on contraction alone, free flooding should occur in all cases, and recur after contraction has passed off.

In the case of a woman delivered with forceps, in the absence of contraction she must inevitably bleed to death. This we know does not happen.

During Cæsarian section the gradual thickening of the uterine wall can be *seen* to be taking place, and that in the absence of contraction.

[1] *Practitioner*, 1907, lxxviii. 361. [2] *Vide* p. 121.

Slight mechanical causes, such as a piece of retained placenta or a full bladder, appear to be able to prevent retraction, and so promote the occurrence of hemorrhage.

G. E. Herman,[1] in continuing previous articles on this subject, points out that Stanmore Bishop, in his arguments against the use of perchloride of iron or other astringents in the treatment of post-partum hemorrhage, is attempting to controvert a proposition put forward by no modern teacher, and states that this method is now extinct. It is a bad method because (1) it is unreliable, and (2) leaves the uterus full of clot, and thus predisposes to sapræmia. He says that no student is taught to ascertain under which of four heads the cause of the hemorrhage falls, and that any student under examination stating that he would search for lacerations with, presumably, speculum, light, and swabs, as his first treatment of post-partum hemorrhage, would be looked upon as a danger to the public, and referred.

Herman refers[2] to the danger of undue haste in securing the termination of the third stage, and says that this arises not from producing "spasmodic and inefficient contraction," as claimed by Bishop, but from the possibility of the membranes not being detached naturally when the expulsion of the placenta takes place, and consequently a piece of chorion being left behind.

Herman does not believe in the numerous causes of post-partum hemorrhage described by Bishop, and states that many of them may be neglected as not being in any way concerned, and knows of no evidence that either drugs or diet during pregnancy have any effect on the amount of bleeding during the third stage of labour.

Herman differs absolutely from Bishop in holding that the current teaching that it is uterine contraction which stops post-partum hemorrhage is correct.

Pressure is only a temporary means of suspending bleeding until the uterus has regained its contractile power.

As regards retraction, Herman reaffirms the definitions of *retraction* and *contraction* practically in the terms generally familiar and almost identical with those used by Fitzgerald, and he states that one great cause of post-partum hemorrhage is the emptying of the uterus in the absence of a pain.

He defines a "tired" uterus as one that is not contracting vigorously, and consequently not doing any harm either to the maternal or fœtal soft parts, and considers that the remedy for this is rest secured by a dose of opium. If, as in the practice of the most experienced accoucheurs, post-partum hemorrhage is caused by the retention of a succenturiate placenta, or piece of membrane in the uterus, the best treatment is to remove it.

He doubts the statement that serious post-postum hemorrhage is caused by cervical lacerations.

[1] *Practitioner*, 1907, lxxviii. 445. [2] *Loc. cit.*

He agrees with Bishop that when with post-partum hemorrhage contraction is absent, pressure is the best thing in the shape of bimanual compression applied to the body of the uterus.

He points out that compression of the aorta in post-partum hemorrhage was advocated by Baudelocque in 1835, and previously by Ramsbotham. Robert Barnes and many other English authors have also recommended it.

In referring to Bishop's suggestion as to the mental state of a surgeon who would squirt hot water at a divided limb to check bleeding, he points out that hot water has a specific contractile effect on the uterus, so that the cases are in no sense parallel.

The objection to an immediate attempt to replace an inverted uterus or remove a piece of placenta, that it is likely to be done with unsterilised hands, is answered by pointing out that if the attendant has done his duty during the second stage, his hands should be sterile, and ready during the third stage for any such emergency.

He objects to the statement that a number of "experiments are to be made"; the measures described are not experiments, but manipulations, each with a definite and necessary object, the final aim being to secure an empty, clean and contracting uterus, with retraction in the intervals between contractions. If contraction cannot be got, pressure must be resorted to. This is best applied to the uterus, but there is no reason why the doctor should not compress the uterus and the nurse the aorta. Lastly, the great prophylactic is, "Do not deliver when the uterus is tired."

This series of articles, in the writer's opinion, confuses the issues. The writers all seem to have some different condition in mind.

Stanmore Bishop, in his advocacy of what is undoubtedly a useful method as an adjunct in severe cases, spoils his case by unnecessary declamation and abuse of an opposition which does not exist.

To suppose that it is necessary to make a prolonged search for lacerations in a case of post-partum hemorrhage is ridiculous. In the first place, as Herman states, hemorrhage from a lacerated cervix is only quite exceptionally severe, and occurs with a hard, contracted uterus, which must be obvious to anyone attending a confinement who observes the condition of the uterus by keeping his hand on it for a reasonable time, not less than half an hour, after the completion of the third stage.

In the second place, if the uterus is watched by the hand on it after the third stage is completed for this period, how often does severe post-partum hemorrhage occur? In the writer's experience, out of close on 8,000 cases, he has met with himself, or been summoned to, only twelve cases, in all of which but two the hemorrhage was stopped by grasping the fundus through the abdominal wall; in these two sudden severe hemorrhage occurred which was stopped by introducing the hand into the uterus and compressing the placental site against the closed fist.

Hemorrhage does not occur even in an inactive uterus so long as the placenta is not detached, and anyone who has done a Cæsarian section knows how firm this attachment is.

Again, in a Cæsarian section, when delivery has been effected in the absence of labour pains, the uterus retracts almost at once after the delivery of the child, and no hemorrhage occurs except from the cut surface, and this ceases directly contraction takes place.

The first method, and the most generally successful one in ordinary cases, is compression of the uterus. Compression of the aorta is a useful adjunct. Elevation of the pelvis, though a most useful proceeding in the treatment of the shock which sometimes succeeds even a slight hemorrhage, is rather a waste of valuable time if compression is omitted. Hot water injection is a valuable stimulant to uterine contraction, but cannot stop hemorrhage in any other way than this, and in addition helps to clear the uterus of clot, etc.

The resort to forceps as soon as signs of exhaustion come on (not because pains are absent), and the management of the third stage without hurry, allowing plenty of time (up to an hour and a half if necessary) for the placenta to be separated naturally while the hand on the fundus guards against and serves as a warning of relaxation, are the measures which are the best means of preventing it.

Ergotin and strychnine, the former in small doses, administered during the last fortnight of pregnancy have been, in the writer's experience, associated with absence of post-partum hemorrhage in cases where it was anticipated, but this, of course, may have been a mere coincidence.

WALTER C. SWAYNE.

---

